# Supplementary material for: Second-Generation Transfer Mediates Efficient Propagation of ICEBs1 in Biofilms
Source: J Bacteriol. 2022 Sep 15;204(10):e00181-22. doi: 10.1128/jb.00181-22 (PMC9578413; doi:10.1128/jb.00181-22)
Supplement: Supplemental file 1 — Fig. S1 and S2, legend of Video S1, and Tables S1 to S3. Download jb.00181-22-s0001.pdf, PDF file, 4.4 MB [file jb.00181-22-s0001.pdf]

A

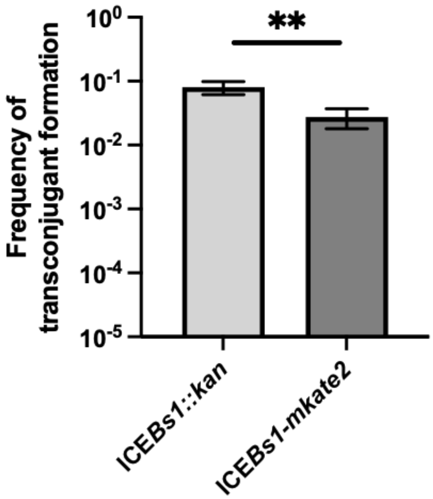

Figure S1. ICEBs1::mkate2 transfer at a slightly lower efficiency than ICEBs1::kan. Donor cells carrying ICEBs1::kan or ICEBs1-mkate2 were mated on MSgg medium at 30 °C for 20 h. The statistical analysis shows that ICEBs1-mkate2 transfer is slightly less efficient than the WT ICEBs1::kan but remains high (t-test; ** P<0.01). Error bars represent the standard deviation (SD). The result is representative of at least three

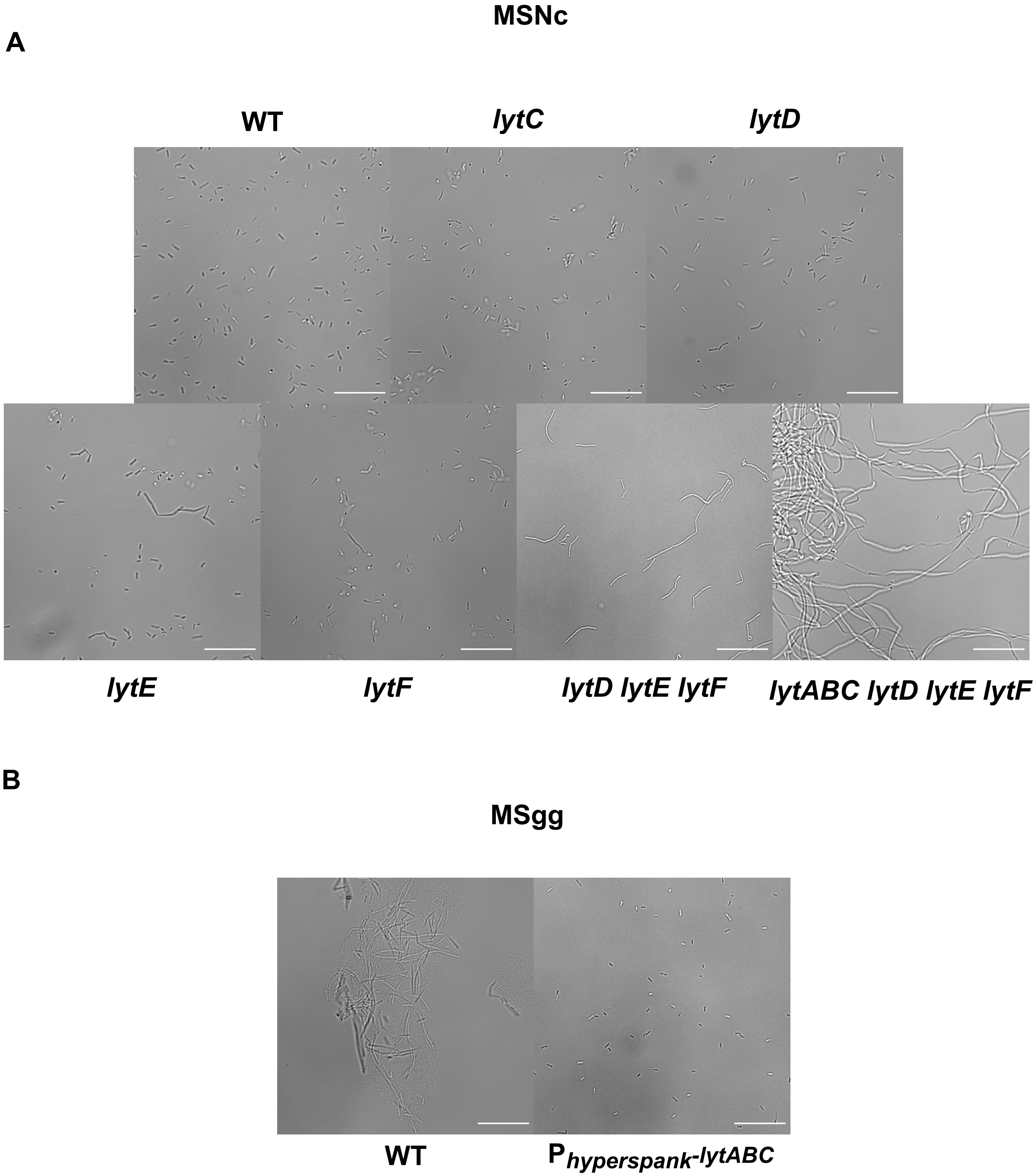

Figure S2. Lytic enzyme mutant phenotype. (A) Lytic mutants were grown in liquid MSNc medium and incubated at 37 °C for 16 h with agitation prior to transferring on an agarose pad and imaged using epifluorescence microscopy. (B) Overexpression of lytABC was grown in liquid MSgg medium containing 50 µM IPTG and incubated at 30 °C for 16 h without agitation prior to transferring on an agarose pad and imaged using epifluorescence microscopy. The scales bars represent a size of 25 µm. Images are representative of the phenotype observed in at least 3 independent replicates°

Video S1. Multiple transconjugant bacteria are not adjacent to donor cells. Donor bacteria bearing ICE*Bs1-mkate2* and expressing a CFP fluorescent protein were mated with recipients expressing a GFP fluorescent protein. Pictures were taken a in Z-stack of 10 µM tick and overlayed using ImageJ 3D Project. The movie shows only donor (blue) and ICE*Bs1* (magenta) to have an easier view of the transconjugants. Donor bacteria appear blue to purple and transconjugant appear bright red. The scale bar represents a size of 10 µm. The video shown is representative of conjugative clusters observed.

Table S1. Strains

Strain	Genotype	Construction
168	**Domesticated strain**	Lab stock
NCIB 3610	**Wild type** Undomesticated strain	Lab stock
JSB86	JH642 ICE*Bs1ΔconG::kan trpC2 pheA1*	This study
JSB93	ICE*Bs1ΔconG::kan*	This study
FL62 (1)	ICEB*s1-kan ycbU-lmrB::spec*	Lab stock
MG25 (1)	ICE*Bs1*^{0} *ylnF/yboA::Tn917 ::amyE::cat*	Lab stock
DL875 (2)	*amyE::Pskf-yfp lacA::PtapA-cfp* (*erm*)	Lab stock
MG13 (1)	ICE*Bs1*^{0}	Lab stock
JSB103	168 *amyE::*$P_{hypersnak}$*-conG* (*spec*)	This study
JSB105	ICE*Bs1*^{0} *amyE::*$P_{hypersnak}$*-conG (spec) lacA::*P_{tapA}*-cfp* (*erm*)	DL875 (2) and JSB103 in MG13 (1)
JSB106	ICE*Bs1ΔconG::kan amyE ::*$P_{hyperspank}$*-conG* (*spec*)	JSB103 in JSB93
CAL51 (3)	JH642 opp::(Tn917lac::pTV21?2 cat) Δ(rapI phrI)342::kan amyE::[(Pspank(hy)-rapI) spc]	Lab stock
MG28 (1)	ICE*Bs1-kan*	Lab stock
JSB107	ICE*Bs1-kan amyE::*$P_{spank(hy)}$*-rapI* (*spec*)	CAL51 (3) in MG28 (1)
VCL28 (1)	*ycbU-lmrB::spec*	Lab stock
JSB97	ICE*Bs1ΔconG::kan ycbU-lmrB::spec*	VCL28 (1) in JSB93
JSB61	168 ICE*Bs1-mkate2* (*spec*)	This study
JSB62	ICE*Bs1-mkate2* (*spec*)	JSB61 in NCIB 3610
JSB68	168 *amyE::*$P_{hyperspank}$*-cfp* (*spec*)	This study
JSB69	ICE*Bs1*^{0} *amyE::*$P_{hyperspank}$*-cfp* (*spec*)	JSB68 in MG13
JSB71	ICE*Bs1-mkate2 amyE::*$P_{hyperspank}$*-cfp* (*spec*)	JSB62 in JSB69
PB69[a]	*amyE ::Physpank-gfp* (cat)	Lab stock
JSB79	ICE*Bs1*^{0} *amyE::*$P_{hyspank}$*-gfp (cat)*	PB69[a] in MG13
PB232[a]	kinD::tet 317amE::kinD WT (spc) lacA::PtapA-yfp (*erm*) ywrK::Tn917::amyE::cat	Lab stock
JSB66	ICE*Bs1-mkate2 lacA::*P_{tapA}*-yfp* (*erm*)	PB232[a] in JSB62
JSB70	ICE*Bs1*^{0} *lacA ::*P_{tapA}*-yfp amyE::*$P_{hyperspank}$*-cfp* (*spec*)	JSB68 and PB232 in MG13 (1)
BJM396[b]	PY79 *lytC::cat*	Lab stock
GJ4	ICE*Bs1*^{0} *lytC::cat*	BJM396[b] in MG13 (1)
BJM402[b]	PY79 *lytD::cat*	Lab stock
GJ5	ICE*Bs1*^{0} *lytD::cat*	BJM402[a] in MG13 (1)

BJM76[b]	PY79 *lytE::cat*	Lab stock
GJ6	ICE*Bs1*^{0} *lytE::cat*	BJM76[b] in MG13 (1)
BJM104[b]	PY79 *lytF::cat*	Lab stock
GJ7	ICE*Bs1*^{0} *lytF::cat*	BJM104[b] in MG13 (1)
L16648	168 *lytABC::neo lytD::tet lytE::cat lytF::spc*	BGSC
BKE09420	*trpC2 ΔlytE::erm*	BGSC
GJ32	ICE*Bs1*^{0} *lytD::tet lytE::erm lytF::cat*	L16648 and BJM104 in MG13 (1)
GJ42	ICE*Bs1*^{0} *lytABC::neo lytD::tet lytE::erm lytF::cat*	L16648 and BJM104 in MG13 (1)
JSB100	168 *amyE::*P$_{hyperspank}$-*lytABC* (*spec*)	This study
JSB101	ICE*Bs1*^{0} *amyE::*P$_{hyperspank}$-*lytABC* (*spec*)	JSB100 in MG13 (1)
FL94 (1)	ICE*Bs1*^{0} *ylnF*/*yboA*::Tn*917*::*amyE*::*cat sinR*::*spec*	Lab stock
PB514[a]	PY79 *ylnF/yloA::Tn917::amyE::cat*	Lab stock
JSB19	ICE*Bs1-kan ylnF/ybaO::tn917::amyE::cat*	PB514[a] in MG28 (1)
SSB569[a]	*tasA::erm epsA-O::tet*	Lab stock
JSB125	ICE*Bs1*^{0} *epsA-O::tet*	SSB569[a] in MG13
PB194[a]	*sinR::spc epsA-O::tet*	Lab stock
JSB127	ICE*Bs1*^{0} *epsA-O::tet sinR::spec*	PB194[a] in JSB125
JSB130	168 *tapA-sinR::erm*	This study
JSB132	ICE*Bs1*^{0} *tapA-sinR::erm*	JSB130 in MG13
JSB133	ICE*Bs1*^{0} *tapA-sinR::erm epsA-O::tet*	JSB130 in JSB125

*Unless indicated, all strains are derivates of *B. subtilis* NCIB 3610. Antibiotic: spectinomycin (spec), chloramphenicol (cat), erythromycin/lincomycin (erm), kanamycin (kan), tetracycline (tet), neomycin (neo)

[a]These strain come from the Kolter and/or Beauregard lab stock.

[b]These strains were gift of the Rudner's Lab

1. Lécuyer F, Bourassa J-S, Gélinas M, Charron-Lamoureux V, Burrus V, Beauregard PB. 2018. Biofilm Formation Drives Transfer of the Conjugative Element ICEBs1 in Bacillus subtilis. mSphere 3:e00473-18.

2. López D, Vlamakis H, Losick R, Kolter R. 2009. Cannibalism enhances biofilm development in bacillus subtilis. Mol Microbiol 74:609–618.

3. Auchtung JM, Lee CA, Monson RE, Lehman AP, Grossman AD. 2005. Regulation of a Bacillus subtilis mobile genetic element by intercellular signaling and the global DNA damage response. Proc Natl Acad Sci U S A 102:12554–12559.

Table S2. Plasmids

Strain	Plasmid name	Genotype	Antibiotics resistance
GJ55	pGJ1	pDR111-*cfp*	ampicillin and spectinomycin
JSB59	pJSB12	pDR111-*yddM-mkate2-attR*	ampicillin and spectinomycin
JSB83	pJSB18	pDR111-*lytABC*	ampicillin and spectinomycin
JSB84	pJSB19	pminiMAD2-*ΔconG*	erythromycin and lincomycin
JSB102	pJSB22	pDR111-*conG*	ampicillin and spectinomycin

Table S3. Primers

Primer	Sequence (5’→3’)
P27	GATCC TTTAA CTCTG GCAAC CCTC
P28	GCCGA CTGCG CAAAA GACAT AATCG
P342	TGTGG ATCCG AATCT CAGAT TGTTA ATCCT GC
P343	TTAGG ATCCA ATCCA GTACA TTACG ATCTC
P344	AATAG GCGAT CGCGG CGAAC TATGA GTTTG CTG
P496	CACTG AGCTC GAGCA ACTTT AGAAA TCGAG TC
P507	GCATG AATTC GACTC TCTAG CTTGA GGCAT C
P508	CATCG CATGC TCATC TGTGC CCCAG TTTG
P719	AGCTG TCGAC TAAAG GAGGA AGATT AGGAG GTGGT TGTAA TG
P673	GCTAG CATGC GCCGA GAATA CCAAA GGAAG AAC
P615	GCCTC CAAGT CTCTA TGTTC AGC
P616	CGAAG CATGC CCAAC TGGTA ATGGT AGCGA C
P647	GTAGG TCGAC ACATA AGGAG GAACT ACTCA TCCTT TGCAC CTCGT CTG
P648	AGATG CATGC GACCT ATGCC AGTCA GTTTA GC
P668	CATCG GATCC AACCA AACGA GCGTG AAGAA G
P669	GTTCT TTCAT CCCTT CTCAG TCGAC AACCA CCTCC TAATC TAACC
P670	GGTTA GATTA GGAGG TGGTT GTCGA CTGAG AAGGG ATGAA AGAAC
P674	GTCCG AATTC ACATG GTCAA CATAA CTTGG GTC
P746	CCAAA TCCGC TATGC AGGAG
P747	GAGGG TTGCC AGAGT TAAAG GATCG GCCAA TCAAT GTCAT CACC
P748	CGATT ATGTC TTTTG CGCAG TCGGC CTGAA AGCTG AAAGA TAAGC AGAAC
P749	AACCG CAAAT AAACG AATAA GGTCC

*Underlined sequence represents restriction site.
